# Identification of Sjögren’s syndrome patient subgroups by clustering of labial salivary gland DNA methylation profiles

**DOI:** 10.1371/journal.pone.0281891

**Published:** 2023-03-02

**Authors:** Calvin Chi, Olivia Solomon, Caroline Shiboski, Kimberly E. Taylor, Hong Quach, Diana Quach, Lisa F. Barcellos, Lindsey A. Criswell

**Affiliations:** 1 Division of Computing, Center for Computational Biology, Data Science and Society, University of California Berkeley, Berkeley, California, United States of America; 2 Genetic Epidemiology and Genomics Laboratory, School of Public Health, University of California Berkeley, Berkeley, California, United States of America; 3 Department of Orofacial Sciences, School of Dentistry, University of California San Francisco, San Francisco, California, United States of America; 4 Department of Medicine, Russell/Engleman Rheumatology Research Center, University of California San Francisco, San Francisco, California, United States of America; 5 Genomics of Autoimmune Rheumatic Disease Section, National Human Genome Research Institute, National Institute of Health, Bethesda, Maryland, United States of America; National Institute of Dental and Craniofacial Research, UNITED STATES

## Abstract

Heterogeneity in Sjögren’s syndrome (SS), increasingly called Sjögren’s disease, suggests the presence of disease subtypes, which poses a major challenge for the diagnosis, management, and treatment of this autoimmune disorder. Previous work distinguished patient subgroups based on clinical symptoms, but it is not clear to what extent symptoms reflect underlying pathobiology. The purpose of this study was to discover clinical meaningful subtypes of SS based on genome-wide DNA methylation data. We performed a cluster analysis of genome-wide DNA methylation data from labial salivary gland (LSG) tissue collected from 64 SS cases and 67 non-cases. Specifically, hierarchical clustering was performed on low dimensional embeddings of DNA methylation data extracted from a variational autoencoder to uncover unknown heterogeneity. Clustering revealed clinically severe and mild subgroups of SS. Differential methylation analysis revealed that hypomethylation at the MHC and hypermethylation at other genome regions characterize the epigenetic differences between these SS subgroups. Epigenetic profiling of LSGs in SS yields new insights into mechanisms underlying disease heterogeneity. The methylation patterns at differentially methylated CpGs are different in SS subgroups and support the role of epigenetic contributions to the heterogeneity in SS. Biomarker data derived from epigenetic profiling could be explored in future iterations of the classification criteria for defining SS subgroups.

## Introduction

Sjögren’s syndrome (SS), increasingly called Sjögren’s disease [1], is a multisystem autoimmune disorder characterized by lymphocytic infiltration of exocrine glands resulting in severe oral and eye dryness, frequent complaints of fatigue and arthralgia, and is the second most common systemic autoimmune disorder in the US, with a female-to-male ratio of 14:1. [2–5]. Currently, the 2016 American College of Rheumatology/European League Against Rheumatism (ACR/EULAR) classification criteria are used to classify SS cases based on the weighted sum of 5 items: anti-SSA(Ro) antibody positivity and FLS with FS ≥1 foci/4mm2, each scoring 3; the ocular staining score (OSS) ≥5, Schirmer test ≤5 mm/5 min, and unstimulated whole saliva (UWS) flow rate ≤0.1 mL/min, each scoring 1. Individuals (with signs/symptoms suggestive of SS) who have a total score ≥4 for the items above, meet the criteria for SS [6]. However, SS remains a heterogenous disease, and no formal criteria exist for further categorizing SS into biologically-relevant disease subgroups. This poses a major challenge for diagnosis, management and therapeutic development for a disease where effective treatment options are limited [2, 7, 8].

Different clinical associations have been observed for different autoantibodies in SS, although the ability of autoantibodies to predict distinct patient subgroups is limited [9]. Recent work utilizing the United Kingdom Primary SS Registry stratified SS cases according to self-reported symptoms of depression, anxiety, pain, fatigue, and dryness using cluster analysis [7]. Tarn *et al*. observed distinct subgroups of SS cases and demonstrated that treatment effects from previously null clinical trials could be detected when case subgroups were considered for re-analysis. However, since this stratification was based on self-reported symptoms, it is unclear to what extent these subgroups reflect underlying pathobiology.

In the current study, we first performed a cluster analysis of DNA methylation data from labial salivary gland (LSG) tissue collected from 131 study participants in the Sjögren’s International Collaborative Clinical Alliance (SICCA) registry [10]. LSG tissue is a prominent target of autoimmune attack in SS and differential methylation between SS cases and non-cases in this tissue is well-established [2, 11–14]. Study participants were comprised of 64 cases and 67 non-cases based upon the 2016 ACR/EULAR classification criteria for SS [6]. Among the participants who met ACR-EULAR criteria, we specifically selected those who had an LSG biopsy with a focus score > = 1 and who had positive serology to anti-(Ro/La)SSA/SSB. Individuals who satisfied only one or no classification criterion items were categorized as non-cases. Cluster analysis involved the agglomerative hierarchical clustering of all study subjects based on low dimensional embeddings of their DNA methylation profiles.

We then investigated the clinical phenotypic characteristics of SS cases that clustered separately. These phenotypic characteristics include outcomes from serological assays, histopathologic examination, oral and ocular tests, and self-reported SS-related symptoms. Finally, we identified regions of differential methylation between SS subgroups and investigated the biological implications of these differences through pathway analysis. The goal of the current study was to identify biologically-relevant disease subgroups of SS that contribute to phenotypic heterogeneity in SS.

## Materials and methods

### Study population and clinical evaluation

Study participants included 64 SS cases and 67 symptomatic non-cases, all of whom were female, non-Hispanic White individuals from the SICCA registry, with well-characterized clinical phenotypic data. Written informed consent was obtained from each participant, and this study was approved by the Institutional Review Board of the Human Research Protection Program at the University of California, San Francisco. The average self-reported age at study entry was 49 years for cases and 46 years for non-cases, with no significant differences (p-value = 0.20). Phenotypic data included salivary, oral, ocular, serological test results, and self-reported symptoms (S1 Table). These self-reported symptoms covered the categories of dryness, fatigue, pain, anxiety, and depression. Eligibility criteria to enroll in the SICCA registry required participants to be 21 years or older and exhibiting at least one of the following: symptoms of dry eyes or dry mouth, prior suspicion/diagnosis of SS, positive serum anti-Ro/SSA, anti-La/SSB, positive rheumatoid factor or an elevated antinuclear antibody titer, sudden increase in dental caries, bilateral parotid gland enlargement [15]. Case status was determined according to the 2016 ACR/EULAR criteria for SS [6]. All cases selected for the current study had an LSG with a positive focus score.

### Methylotyping and preprocessing

LSGs collected as part of the SICCA project were flash-frozen, and stored in liquid nitrogen following standardized procedures at time of enrollment. While the SICCA project included 9 research sites, across which all data collection was standardized through training and calibration protocols involving all sites, LSGs included in the current study were collected on participants enrolled at one site only, the UCSF site, which was the only SICCA site to also collect peripheral blood mononuclear cells (PBMCs) [10, 16]. Whole LSG (one per participant) of similar sizes were processed for DNA extraction using a standardized protocol. DNA methylation was measured for each LSG using the Illumina 450K Infinium Methylation BeadChip (450K) platform for 28 LSGs and the Infinium MethylationEPIC (EPIC) platform for 103 LSGs. The 450K and EPIC chips allow for high-throughput interrogation of more than 450,000 and 850,000 highly informative CpGs sites respectively, spanning ~22,000 genes across the genome.

Methylation data processing was performed using *Minfi*, a Bioconductor package for the analysis of Infinium DNA methylation microarrays [17]. Background subtraction with dye-bias normalization was performed on methylated and unmethylated signals with the “noob” procedure, followed by quantile normalization with *preprocessQuantile* [18].

For joint analysis of all 131 LSG samples, the intersection of CpGs from the 450K and EPIC chips were selected for analysis, resulting in an initial set of 452,832 CpGs. Probes where more than 5% of samples had a detection p-value > 0.01 were removed, to retain probes where signal is distinguishable from negative control probes. To remove probes with ambiguous methylation measurements due to incomplete binding between DNA strand and probe, probes with SNPs with minor allele frequency greater than 0% at either the probe site, CpG interrogation site, or single nucleotide extension were removed. Finally, cross-reactive probes, or probes with probe-binding specificity and polymorphic targets problems, were removed [19]. The final preprocessed dataset consisted of 336,040 CpG sites. Since no subject had more than 5% of probes with detection p-value > 0.01, all 131 subjects were retained.

Both methylation measures of β-values and M-values were used for this study. A β-value is the ratio of the methylated probe intensity to the sum of methylated and unmethylated probe intensities, and reflects the proportion of methylation at a CpG site. The M-value can be derived from a β-value as ${\text{log}}_{2}\frac{\beta }{1-\beta }$, and was used for identifying differentially methylated regions (DMRs) due to less severe heteroscedasticity [20].

### Genotyping and quality control

The participant genotypes were obtained from the larger SICCA cohort, which was genotyped on the Illumina HumanOmni2.5-4v1 or Illumina HumanOmni25M-8v1-1 arrays from DNA extracted from whole blood. All quality control steps performed have been previously described [21]. The final genotype dataset consisted of 1,392,448 SNPs. Multidimensional scaling (MDS) was performed on all genotype data along with reference European subpopulations from the Human Genome Diversity Project (HGDP) [22], to confirm self-reported non-Hispanic white individuals were of European ancestry. There were no genetic ancestry differences between cases and non-cases *(data not shown)*.

### Removing unwanted DNA methylation variation

Since LSGs were methylotyped on both the 450K and EPIC chip, we adjusted for batch effects due to array type (S1 Fig). We applied parametric empirical Bayes using *ComBat* from the *SVA* package to adjust β-values for array type [23]. Since *ComBat* requires no missing values, missing methylation values were mean imputed per CpG site before adjustment, then missingness restored afterwards.

### Variational autoencoder summary

We used a variational autoencoder (VAE) to perform a non-linear projection of methylation data onto a low dimensional latent space. The VAE achieves this by mapping input data to a distribution of latent variables whose samples are used to reconstruct the input data [24]. The VAE is comprised of an encoder that estimates the parameters of the latent variable distribution, and a decoder that attempts to reconstruct the data from the latent features. The encoder and decoder are typically parameterized by neural networks acting as effective function approximators. See S1 File for additional details.

### Hierarchical clustering

All clustering was performed using agglomerative hierarchical clustering with Ward’s minimum variance method as the link function [25]. At each merge iteration in hierarchical clustering, Ward’s method merges the pair of clusters that leads to the minimum increase in total within-cluster variance after merging. Similar to other link functions such as complete linkage, Ward’s method tends to produce more balanced dendrograms and is less sensitive to outliers. Euclidean distance between latent features is used for hierarchical clustering in the latent space of DNA methylation data. In contrast, the baseline hierarchical clustering method uses the average absolute difference in β-values to compare a given pair of individuals. When clustering was based on differences in β-values instead of VAE embeddings, results were similar (S2 Fig), with about 9% of participants who clustered differently. The clusters studied in this paper were based on DNA methylation embeddings.

### Statistical testing

The Wilcoxon Rank Sum test was used to test the difference in ordinal or continuous clinical phenotypes between cases across patient clusters, and the Kruskal-Wallis test was used to test the difference between four participant clusters when appropriate. The chi-square test of independence was used to test association between categorical clinical variables (i.e., nominal and dichotomous) and patient clusters or disease subgroup.

Since focus score is only computed when lymphocytic foci are present (i.e., when a diagnosis of focal lymphocytic sialadenitis is made), the focus score was set at zero for the purpose of statistical analysis for participants whose LSG biopsy diagnosis was within normal limits, non-specific chronic inflammation, or sclerosing chronic sialadenitis. No other phenotype analyzed had more than two missing values, and missing values were omitted from statistical tests. Tear break-up times of greater than or equal to 10 seconds were considered healthy, so these times were truncated and set to 10 seconds.

### Identification of differentially methylated regions

DMRs were identified using *bumphunter*, which identifies regions of CpG sites which are all hypermethylated or hypomethylated in one group of subjects compared to the other [26]. In this study, a candidate DMR was required to have at least two CpG sites and have an effect size of at least 1.0, where the effect size is the expected change in methylation from one group to the other. The linear regression specified for *bumphunter* was

M~outcome+arraytype, (1)

controlling for array type. Here “M” is the M-value without batch correction, *array type* is an indicator variable for whether a subject was methylotyped on 450K, and *outcome* is whether the subject belongs to the “severe” or “mild” disease subgroup as defined in our Results following clustering analysis. The number of permutations was set at *B* = 1,000 for generating a null distribution of candidate DMRs for establishing significance, with *nullMethod* = “bootstrap” to control for the adjustment covariate. Significant DMRs were stringently selected as those with *fwerArea* ≤ 0.05, defined as proportion of permutations with maximum bump area greater than the observed area for a DMR. *Minfi* was used to annotate each DMR with its nearest gene in base pairs, location relative to nearest gene, and location relative to nearest CpG island. Detailed descriptions of each DMR gene available from National Center for Biotechnology Information were obtained with Biopython [27].

### Gene set enrichment analysis

We restricted GSEA to genes differentially methylated at the promoter or gene body, since differential methylation at these regions has been shown to regulate gene expression [28]. To provide a qualitative picture of the biological processes impacted by differential methylation, DMR genes were tested for enrichment of gene ontology (GO) gene sets from the Molecular Signatures Database combined with SS-related gene sets from past studies using the hypergeometric test [13, 29, 30]. False discovery rate was controlled with the Benjamini-Hochberg procedure [31]. GSEA was performed separately for hypermethylated and hypomethylated DMR genes.

## Results

### Identification of study participant clusters

**Fig 1:**
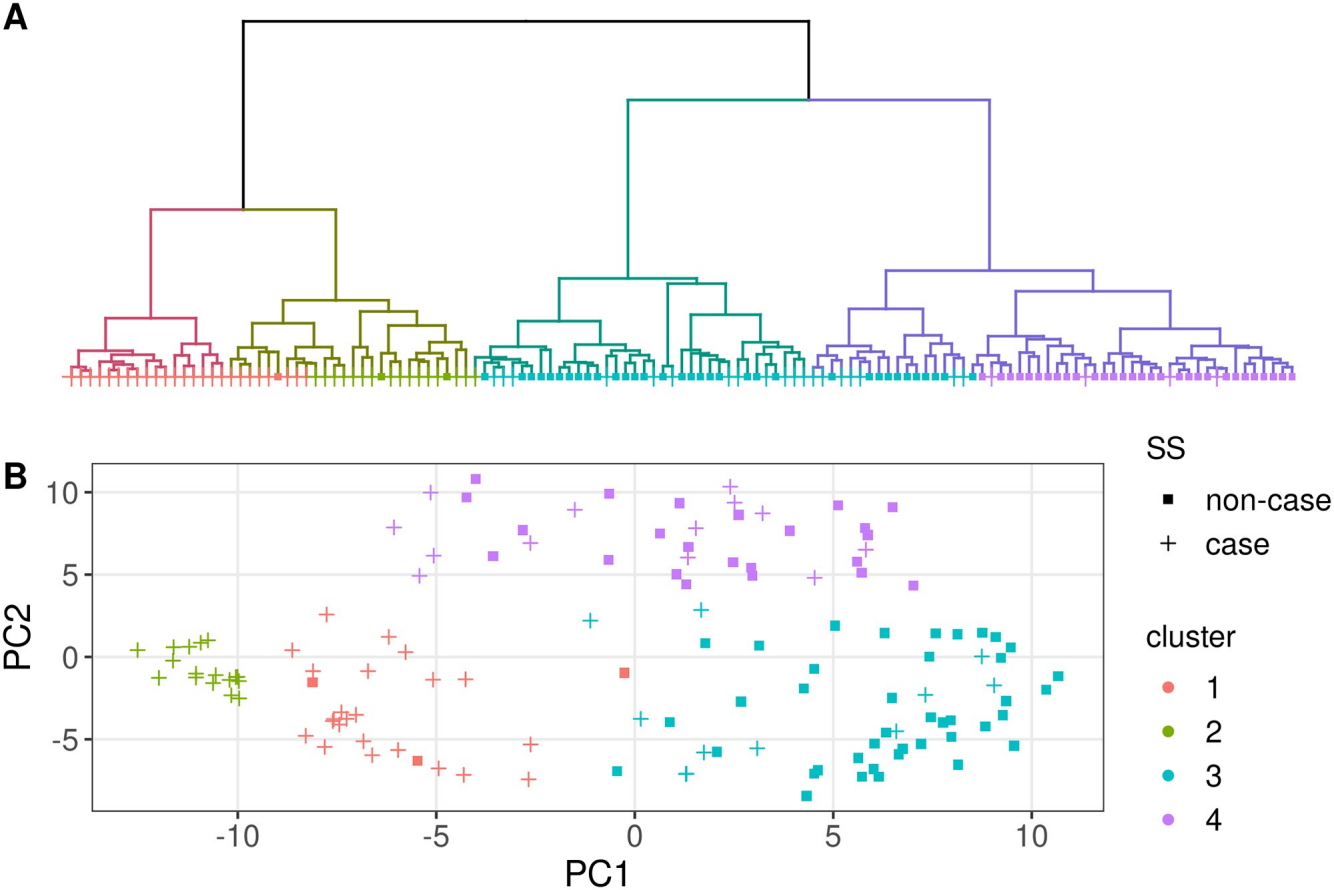
Clustering analysis of LSG (labial salivary gland) DNA methylation profiles. (A) Dendrogram from hierarchical clustering of VAE-based low dimensional embeddings from all 131 participants, with cluster numbering in the bottom and dendrogram cut height indicated by horizontal dotted line. (B) PCA plot of VAE-based low dimensional embeddings for all 131 study participants, with cluster numbering denoted by color and SS status denoted by shape.

Hierarchical clustering of VAE-based low dimensional embeddings of DNA methylation profiles identified four clusters within the study sample of 131 participants. Clusters 1 and 2 exhibited the greatest intercluster distance from clusters 3 and 4 (Fig 1A). A principal components analysis (PCA) plot of these embeddings further visualized this clustering pattern (Fig 1B). Clusters 1 and 2 are “SS-dominant” with 93.0% of participants being SS cases, and clusters 3 and 4 are “non-case dominant” with 72.7% of subjects being non-cases. Despite a high concordance between the observed clusters based on DNA methylation data and case status (p-value = 2.4 x 10^−11^), about 37% of SS cases in our sample clustered with non-cases in clusters 3 and 4. Across clusters, we did not detect any significant differences in history of cigarette use (p-value = 0.72), cigarettes smoked per day (p-value = 0.17), self-reported age of SS onset (p-value = 0.29), or anticholinergic drug use (p-value = 0.18), which have all been shown to influence DNA methylation [32–34]. A summary of potential confounders between cases versus non-cases and severe versus mild cases (as defined below) can be found in S2 and S3 Tables, respectively. Additional sensitivity analyses clustering within only SS cases did not meaningfully change cluster membership with 95% of subjects remaining in the same cluster. Cluster assignment of study subjects are provided in S4 Table.

### Clinical phenotype comparisons between clusters

A comparison of clinical variables capturing serologic status, histopathologic findings, and oral and ocular test results showed that participants in clusters 1 and 2 on average had more severe phenotypes compared to subjects in clusters 3 and 4 (Table 1); evidence for clinical heterogeneity across clusters was observed (p-value < 0.05). Differences in phenotype severity was expected, since many of these clinical measures serve as the basis for the 2016 ACR/EULAR SS criteria [6], and clusters 1 and 2 are SS-dominant compared to clusters 3 and 4. However, with the exception of mouth pain (p-value = 0.01), no significant differences in self-reported symptoms related to dryness, fatigue, pain, anxiety, or depression were observed between the four clusters (Table 1, S5 Table; S3 Fig). In each cluster, a majority of study participants reported dryness of the mouth, eyes, and vagina. The self-reported severities based on scoring for the other symptom categories of fatigue, pain, anxiety, and depression were low overall (S3 Fig).

**Table 1:** Clinical phenotype analysis by cluster.

Variable	Cluster 1	Cluster 2	Cluster 3	Cluster 4	p-value
	(n = 26)	(n = 17)	(n = 52)	(n = 36)	
**Antinuclear antibody at 1:320 concentration level, %**	73.08	94.12	19.23	30.56	**3.94E-09**
**Immunoglobulin G, mg / dL (SD)**	1370.31 (480.31)	2130.53 (597.66)	995.69 (295.31)	1222.33 (851.38)	**1.48E-08**
**Complement Component 3, mg / dL (SD)**	117.65 (17.66)	118.71 (30.55)	122.06 (33.20)	128.42 (23.88)	1.89E-01
**Complement Component 4, mg / dL (SD)**	24.42 (7.20)	22.00 (9.88)	26.92 (9.10)	27.89 (8.15)	**2.81E-02**
**Anti-La/SSB +, %**	50.00	94.12	5.77	8.33	**6.38E-14**
**Rheumatoid Factor result, %**	65.38	94.12	13.46	16.67	**2.33E-11**
**Tear break-up time left type, seconds (SD)**	4.88 (3.17)	4.53 (2.83)	7.71 (2.80)	6.11 (3.19)	**1.45E-04**
**Tear break-up time right type, seconds (SD)**	4.88 (2.94)	3.59 (2.32)	7.52 (2.85)	5.58 (2.98)	**7.27E-06**
**Unstimulated whole salivary flow rate, ml / 5 min (SD)**	0.42 (0.51)	0.19 (0.21)	0.67 (0.50)	0.55 (0.52)	**3.80E-04**
**Focus score (SD)**	2.97 (1.85)	4.77 (2.04)	0.52 (1.02)	1.13 (1.48)	**1.64E-13**
**max(ocular SICCA score left eye, ocular SICCA score right eye) (SD)**	7.58 (2.89)	9.18 (2.79)	4.33 (2.95)	4.54 (3.35)	**1.46E-07**
**Right parotid gland enlargement, %**	7.69	17.65	3.85	0.00	5.42E-02
**Left parotid gland enlargement, %**	7.69	17.65	5.77	2.78	2.45E-01
**Dry mouth symptoms, %**	88.46	100.00	98.08	88.89	1.33E-01
**Needs liquids for swallowing, %**	84.62	94.12	65.38	69.44	5.75E-02
**Dry eye symptoms, %**	92.31	94.12	88.46	91.67	8.80E-01
**Lymphoma, %**	0.00	0.00	0.00	0.00	NA
**Presence of germinal center-like formation tested with H&E staining, %**	19.23	29.41	0.00	0.00	**2.93E-05**
**Satisfies 2016 ACR/EULAR SS criteria** [3]					
**LSG with focal lymphocytic sialadenitis and focus score ≥ 1, %**	88.46	100.00	23.08	41.67	**1.73E-10**
**Anti-Ro/SSA +, %**	61.54	94.12	5.77	13.89	**8.52E-14**
**Ocular staining score ≥ 5 on at least one eye, %**	88.46	94.12	51.92	47.22	**1.08E-04**
**Schirmer ≤ 5 mm/5min on at least one eye, %**	11.54	41.18	11.54	11.11	**1.74E-02**
**Unstimulated whole saliva flow rate ≤ 0.1 ml/min, %**	76.92	94.12	46.15	50.00	**7.32E-04**
**SS, %**	88.46	100.00	21.15	36.11	**2.43E-11**

Clinical phenotype averages by patient cluster, determined from the VAE-based clustering analysis. P-values were computed using Kruskal-Wallis test for ordinal or continuous phenotypes, and chi-square test of independence for categorical or binary phenotypes. Significant p-values at *α* = 0.05 are bolded. Note the average is equivalent to proportion for binary phenotypes.

**Fig 2:**
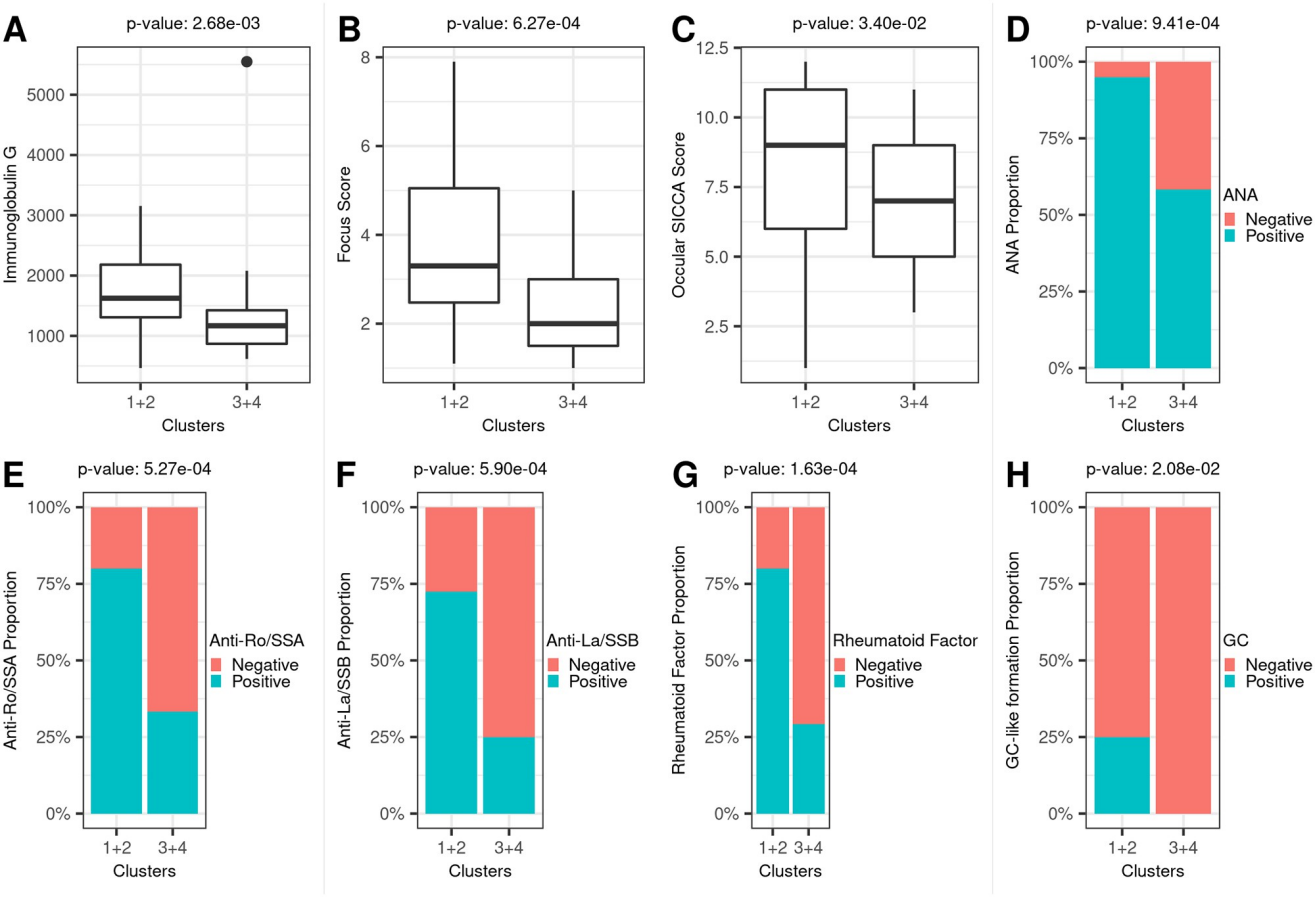
Clinical phenotype comparison between severe SS and mild SS. Plots of clinical phenotypes that are different at an α = 0.05 significance level between severe SS cases and mild SS cases. Severe SS cases are from clusters 1 and 2 and mild SS cases are from clusters 3 and 4. P-values from Wilcoxon rank sum tests or chi-square tests of independence are shown above each subplot. (A) Immunoglobulin G box plot. (B) Focus score box plot. (C) Ocular SICCA score (maximum of left and right eyes) box plot. (D) Detection of antinuclear antibody at 1:40 concentration level bar plot. (E) Anti-Ro/SSA bar plot. (F) Anti-La/SSB bar plot. (G) Rheumatoid factor bar plot. (H) Germinal center (GC)-like formation bar plot.

We next compared SS cases in clusters 1 and 2 (n = 40) against SS cases in clusters 3 and 4 (n = 24) to characterize heterogeneity in clinical phenotype patterns between the two SS case subgroups. We observed evidence for increased clinical phenotype severities at an α = 0.05 significance level in SS cases from clusters 1 and 2 compared to SS cases from clusters 3 and 4 (Table 2, Fig 2). Specifically, a greater proportion of SS cases in clusters 1 and 2 were positive for anti-Ro/SSA, anti-La/SSB, rheumatoid factor, and germinal center-like structures on LSG biopsies. SS cases from clusters 1 and 2 also had higher titers of antinuclear antibodies, higher levels of immunoglobulin G, higher ocular SICCA scores [35], and higher focus scores (Table 2, Fig 2). Based on these results, herein we refer to the SS cases from clusters 1 and 2 as “severe SS” and cases from clusters 3 and 4 as “mild SS”. No significant differences in self-reported symptoms or the prevalence of physician-confirmed, SS-related extraglandular manifestations were present between severe SS and mild SS (Table 2, S6 Table).

**Table 2:** Clinical phenotype analysis by disease subgroup.

Variable	Mild SS (n = 24)	Severe SS (n = 40)	p-value
**Antinuclear antibody at 1:320 concentration level, %**	45.83	85.00	**2.38E-03**
**Immunoglobulin G, mg / dL (SD)**	1353.04 (975.22)	1716.55 (644.92)	**2.68E-03**
**Complement Component 3, mg / dL (SD)**	131.33 (28.85)	118.10 (23.18)	8.29E-02
**Complement Component 4, mg / dL (SD)**	25.63 (7.26)	23.30 (8.61)	5.70E-02
**Anti-La/SSB +, %**	25.00	72.50	**5.90E-04**
**Rheumatoid Factor result, %**	29.17	80.00	**1.63E-04**
**Tear break-up time left type, seconds (SD)**	6.04 (3.30)	4.60 (3.00)	9.94E-02
**Tear break-up time right type, seconds (SD)**	5.29 (3.14)	4.28 (2.75)	1.97E-01
**Unstimulated whole salivary flow rate, ml / 5 min (SD)**	0.43 (0.46)	0.29 (0.34)	4.36E-01
**Focus score (SD)**	2.44 (1.23)	3.96 (1.91)	**6.27E-04**
**max(ocular SICCA score left eye, ocular SICCA score right eye) (SD)**	7.04 (2.51)	8.38 (2.78)	**3.40E-02**
**Right parotid gland enlargement, %**	4.17	12.50	5.06E-01
**Left parotid gland enlargement, %**	4.17	12.50	5.06E-01
**Dry mouth symptoms, %**	95.83	92.50	1.00E+00
**Needs liquids for swallowing, %**	70.83	90.00	1.04E-01
**Dry eye symptoms, %**	95.83	92.50	1.00E+00
**Lymphoma, %**	0.00	0.00	NA
**Presence of germinal center-like formation tested with H&E staining, %**	0.00	25.00	**2.08E-02**
**Satisfies 2016 ACR/EULAR SS criteria** [3]			
**LSG with focal lymphocytic sialadenitis and focus score ≥ 1, %**	100.00	100.00	NA
**Anti-Ro/SSA +, %**	33.33	80.00	**5.27E-04**
**Ocular staining score ≥ 5 on at least one eye, %**	79.17	92.50	2.42E-01
**Schirmer ≤ 5 mm/5min on at least one eye, %**	20.83	25.00	9.39E-01
**Unstimulated whole saliva flow rate ≤ 0.1 ml/min, %**	66.67	85.00	1.60E-01

Clinical phenotype averages for severe SS cases and mild SS cases. Severe SS cases belong to clusters 1 and 2 and mild SS cases belong to clusters 3 and 4 from the VAE-based clustering analysis. P-values were computed using Wilcoxon rank sum test for ordinal or continuous clinical phenotypes, and computed using chi-square test of independence for categorical or binary phenotypes. Significant p-values at *α* = 0.05 are bolded. Note the average is equivalent to proportion for binary phenotypes.

We also investigated whether severe and mild SS cases scored differently on the individual 2016 ACR/EULAR classification criteria items [6]. While all subjects satisfy the 2016 ACR/EULAR classification criteria to be considered SS cases, the proportion of severe SS cases who satisfy the anti-Ro/SSA criterion, specifically, was two times higher than the proportion of mild SS cases (p-value = 5.27E-4). Saliva flow rate, Schirmer’s test, ocular staining score, and focus score proportions were similar between the severe and mild SS case subgroups (Table 2). Although all SS cases satisfy the focus score criterion, significantly higher scores were observed in severe SS cases (average focus score = 3.96) compared to mild SS cases (average focus score = 2.44) (Table 2).

#### Differentially methylated regions between disease subgroups

We identified DMRs that distinguish severe from mild SS cases. Overall, we observed significant hypomethylation at the MHC region and hypermethylation in other areas of the genome (S4A Fig). We identified a total of 207 significant DMRs from 826 candidate DMRs, with 41 hypomethylated regions and 166 hypermethylated regions, in severe SS cases relative to mild SS cases (S8 Table).

**Fig 3:**
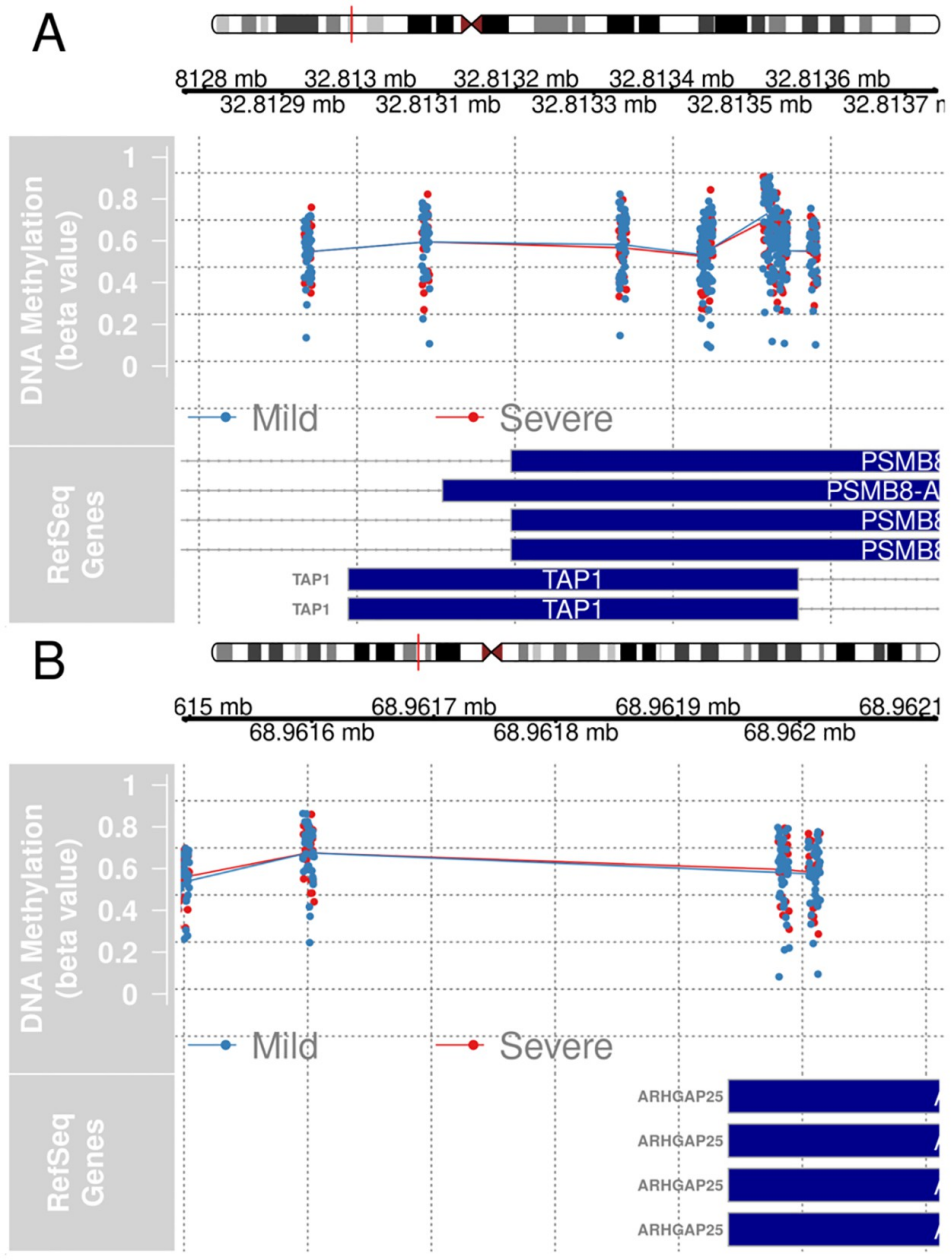
Statistically significant DMRs (*fwerArea* ≤ 0.05) between severe and mild SS cases located in genes previously identified as hypomethylated in cases relative to non-cases. (A) DMR located in *PSMB8* and *TAP1*, (B) DMR located in *ARHGAP25*.

Gene set enrichment analysis (GSEA) of hypomethylated genes revealed an overall enrichment of immune-related biological processes (Table 3). The hypomethylated genes *AIM2*, *CTSZ*, *PSMB8*, *TAP1*, *LCP2*, and *ARHGAP25* were previously identified as hypomethylated in SS cases relative to non-cases (Fig 3.) [13]. Similar differential methylation patterns were observed for these genes in a comparison of severe SS cases to mild SS cases. Some of the other top enriched biological processes are known to be involved in the pathobiology of SS, such as response to type I interferon and T cell migration [36]. For GSEA of hypermethylated genes, many neurological processes appeared in the top 10 results (S7 Table). Another top enriched gene set was the regulation of cell fate commitment, which could potentially reflect differences in proportion of immune cells that infiltrated the LSG in SS patients [37].

**Table 3:** Top gene sets enriched for hypomethylated genes.

Gene set	n	Overlap genes	p-value	adj. p-value
SS DMP genes	6	*AIM2*, *CTSZ*, *PSMB8*, *TAP1*, *LCP2*, *ARHGAP25*	5.21E-13	2.35E-9
Response to Type I interferon	5	*HLA-E*, *XAF1*, *PSMB8*, *ISG20*, *IRF5*	2.04E-10	4.60E-7
Mast cell activation	3	*PIK3CD*, *LCP2*, *RHOH*	1.00E-8	1.50E-5
Cellular extravasation	3	*PIK3CD*, *TNF*, *ITGB2*	2.11E-8	2.38E-5
Positive regulation of monooxygenase activity	3	*TNF*, *GDNF*, *NPR3*	3.96E-8	3.03E-5
Tumor necrosis factor receptor binding	3	*TNFSF13B*, *TNF*, *TRAF3*	4.56E-8	3.03E-5
Receptor metabolic process	4	*GRB2*, *TNF*, *ITGB2*, *CD81*	4.72E-8	3.03E-5
Antigen processing and presentation of peptide antigen via MHC class I	4	*HLA-E*, *PSMB9*, *PSMB8*, *TAP1*	7.98E-8	4.48E-5
Spleen development	3	*PSMB9*, *PITX2*, *PKN1*	1.36E-7	6.78E-5
Regulation of immunoglobulin production	3	*HLA-E*, *TNF*, *PKN1*	2.92E-7	1.10E-4

Gene set enrichment analysis of genes near DMRs that are hypomethylated in severe SS cases relative to mild SS cases. Candidate gene sets include GO gene sets from the Molecular Signatures Database [29], genes known to harbor differentially methylated CpG sites between SS cases and non-cases (SS DMP genes) [13], and genes known to be differentially expressed between SS cases and healthy controls (SS DE genes) [30]. n = number of overlapping genes; adj. p-value = Benjamini-Hochberg adjusted p-value.

For the majority of variable CpG sites, the four clusters (cases and non-cases) had distinct levels of DNA methylation. Principal component analysis (PCA) of methylation embeddings showed that clusters were roughly ordered as cluster 2, cluster 1, cluster 4, and cluster 3 on PC1 (Fig 1B). This ordering corresponded to that by phenotype severity; each cluster had distinct levels of DNA methylation globally which was correlated with phenotype severity. Our analysis of average DMR methylation levels at the MHC supported this interpretation. S4B Fig shows that the cluster ordering from most to least hypomethylated is cluster 2, cluster 1, cluster 4, and cluster 3, which is the same ordering as that based on PC1. Looking at PC2 (Fig 1B), cluster 4 is separated from the other clusters, suggesting subjects in cluster 4 have CpG sites which are uniquely differentially-methylated compared to the rest of the subjects.

## Discussion

In the current study, our results revealed clinically meaningful clusters based on DNA methylation profiling of LSGs that differ from classification by the 2016 ACR/EULAR SS criteria. Specifically, we identified two subgroups of SS cases with significantly different LSG DNA methylation levels and risk allele frequencies, both at the MHC. These biological differences are strongly associated with clinical phenotype severity, with one subgroup consistently experiencing greater severity across phenotypes compared to the other. We refer to these two subgroups of cases as severe SS and mild SS. While many mild SS cases clustered with non-cases, very few non-cases clustered with severe SS cases. Our findings may have implications for SS classification, but are also potentially relevant to SS management and therapeutic development.

Phenotypic comparisons between SS cases in clusters 1 and 2 versus SS cases in clusters 3 and 4 showed that individuals in clusters 1 and 2 have higher focus scores, germinal center-like formations, ocular staining scores, and a higher frequency of autoantibodies. Thus, SS cases in clusters 1 and 2 are considered severe SS, and cases in clusters 3 and 4 are considered mild SS. In contrast, self-reported symptoms did not differ significantly between the two SS case subgroups. Since severe SS cases also have greater hypomethylation, this suggests a functional link with autoantibody production differences between the SS subgroups; further studies are needed to elucidate these contributions to SS. MHC associations with autoantibody manifestations have been demonstrated in both SS and systemic lupus erythematosus in Europeans in case-control studies [38–40]. The four symptom score patterns observed by Tarn *et al*. [7] (e.g. pain dominant with fatigue) were not observed in our study. This lack of correspondence could be due to a smaller sample size in this study and also to the inclusion of non-cases.

Through clustering based on LSG DNA methylation profiles, we found that SS cases in clusters 3 and 4 tended to have LSG DNA methylation levels that were more similar to those of non-cases. With the exception of self-reported symptoms, these SS cases had more mild clinical phenotypes compared to SS cases in clusters 1 and 2. DMR analysis revealed a general hypomethylation at the MHC region and immune-related genes (Table 3). This pattern was previously observed by Cole *et al*. in LSG [13], in a case-control study. While some subjects in the current study overlapped with those in our previous study, the current study is much larger and focused primarily on SS cases. Findings support the role of epigenetic contributions to heterogeneity in SS. We report for the first time that these differences are associated with severity for many key clinical phenotypes.

Currently, the 2016 ACR/EULAR classification criteria do not distinguish between the severe and mild SS case subgroups identified in the current study based on LSG DNA methylation profiles. Since LSG biopsies are already used for SS classification [2], LSG methylation profiling may be a convenient approach to further characterize SS case subgroups. DNA methylation assays have been used in clinical practice to aid in the detection, treatment, and monitoring of cancers [41]. If epigenetic therapy becomes an effective treatment for SS, our results have implications for targeting the most relevant disease subgroup, due to differences in epigenetic profiles. Studies suggest that this approach represents a promising direction for the treatment of autoimmune diseases such as SS [42, 43].

Based on our phenotype analyses, severe SS cases may be at higher risk of lymphoma than mild SS cases due to a higher frequency of lymphoma risk factors [2] (Table 2). Specifically, these risk factors include the swelling of parotid glands, presence of rheumatoid factor, a lower C4 level, a higher focus score, and the presence of germinal center-like formations based on hematoxylin and eosin staining. Of these, severe SS cases have significantly higher presence of rheumatoid factor, focus scores, and presence of germinal center-like formation compared to mild SS cases (Table 2). However, there were no cases of physician confirmed lymphoma in SS cases (Table 2), nor were there significant differences in the prevalence of other physician confirmed, SS-related extraglandular manifestations (i.e., thyroid, liver, kidney, and any other systemic disease) between the clusters (S5 Table) [15]. Future work should include an examination of lymphoma risk in SS using larger sample sizes.

Our results raise some questions regarding the underlying biology of SS case subtypes and how to properly classify them. Since the LSG consists of a mixture of epithelial and inflammatory cells, one study limitation is that it is not yet clear the extent to which DNA methylation differences reflect differences in the composition of cellular infiltration. The mean focus score, which is a measure of inflammatory infiltrates, corresponded though not perfectly, with reduced hypomethylation in the observed clusters. Previous observations of differentially methylated cell differentiation markers in LSG and enrichment of the regulation of cell fate commitment gene set in our study support the “tissue-heterogeneity interpretation” [13, 37] (S7 Table). On the other hand, cell-specific differential methylation has been observed for salivary gland epithelial cells in SS [14]. A strength of DNA methylation profiling in the current study is that results support the identification and characterization of genes and pathways involved in SS pathogenesis. Another question is whether there is a causal relationship between genetic variation and DNA methylation at the MHC and SS cases subgroups. While we observed that many genes involved in neurological processes are hypermethylated (S7 Table), the pathological mechanism by which SS leads to the damage of the nervous system is not well-established, but it is thought to involve inflammatory infiltration of the dorsal root ganglia [2, 44, 45]. Our identification of biological subtypes of SS raises the question of whether the 2016 ACR/EULAR classification criteria could be revised in the future to include additional molecular data derived from genetic and/or epigenetic profiling. A recent study has shown that In addition to LSG tissue, differential DNA methylation between SS cases and controls has also been shown in CD4+ T cells, CD19+ B cells, and whole blood [12, 46–48]. While there is evidence to suggest that LSG removal does not lead to significant morbidity in SS cases [49, 50], if epigenetic profiles from whole blood can provide similar disease subtype information as LSG tissue, then more efficient ways of using DNA methylation for disease classification may be feasible. DNA methylation arrays could be utilized in a clinical setting, similar to SNP arrays [51], and in combination with SNP arrays to improve phenotype classification as recently described [52].

## Supporting information

S1 Fig PCA of β-values with and without batch-correction. The DNA methylation array (i.e. 450K or EPIC) is indicated by color. PC2 captures variation in DNA methylation explained by array type. (A) Before batch-correction. (B) After batch-correction.
(TIF)

S2 Fig Baseline clustering approach. (A) Dendrogram of baseline hierarchical clustering of DNA methylation profiles (see Materials and methods). (B) Confusion matrix showing agreement of clustering results between the baseline approach and VAE-based approach (Fig 1).
(TIFF)

S3 Fig Heatmap of self-reported SS symptoms. All phenotypes are either ordinal or binary, and normalized between 0 and 1, with larger values indicative of greater severity. Clinical phenotypes are grouped by general categories of dryness, fatigue, pain, anxiety, and depression. Each column represents a patient and all 131 subjects are grouped by patient clusters. Gray indicates missingness. See S4 Table for clinical phenotype key.
(TIFF)

S4 Fig Differential methylation at the MHC. Dot plot of average β-value over DMR CpG sites at the MHC region, by (A) SS severity (0 = mild, 1 = severe) and (B) cluster. Each dot represents an individual’s average β-value at the MHC.
(TIFF)

S5 Fig VAE training and validation loss. (TIFF)

S1 Table Demographic and clinical phenotype variables. (XLSX)

S2 Table Demographic and clinical variables compared in cases versus non-cases. (XLSX)

S3 Table Demographic and clinical variables compared in mild versus severe cases. (XLSX)

S4 Table Cluster assignment of study subjects. The column “geoID” contains subject IDs of methylation data in GEO (accession number GSE166373), the column “dbgapID” contains subject IDs of genotypes, demographic variables, and clinical data in dbGaP (accession number phs000672.v1.p1), the column “cluster” contains cluster assignment, and the column “SS” contains SS case status.
(XLSX)

S5 Table Analysis of SS-related symptoms, by cluster. Averages of self-reported SS symptoms, by patient cluster, determined from VAE-based clustering analysis. P-values were computed using Kruskal-Wallis test for ordinal or continuous clinical phenotypes, and computed using chi-square test of independence for categorical or binary phenotypes. Refer to S3 Table for key of clinical phenotype abbreviations. Note the average is equivalent to proportion for binary phenotypes.
(XLSX)

S6 Table Analysis of SS-related symptoms, by disease subgroup. Averages of self-reported SS symptoms for severe SS cases and mild SS cases. Severe SS cases belong to clusters 1 and 2 and mild SS cases belong to clusters 3 and 4 from the VAE-based clustering analysis. P-values were computed using Wilcoxon rank sum test for ordinal or continuous clinical phenotypes, and computed using chi-square test of independence for categorical or binary phenotypes. Refer to S3 Table for key of clinical phenotype abbreviations. Note the average is equivalent to proportion for binary phenotypes.
(XLSX)

S7 Table Top gene sets enriched for hypermethylated genes. Gene set enrichment analysis of genes near DMRs that are hypermethylated in severe SS cases relative to mild SS cases. Candidate gene sets include GO gene sets from the Molecular Signatures Database (30), genes known to harbor differentially-methylated CpG sites between SS cases and non-cases (SS DMP genes) (9), and genes known to be differentially expressed between SS cases and healthy controls (SS DE genes) (57). *n* = number of overlapping genes; adj. p-value = Benjamini-Hochberg adjusted p-value.
(XLSX)

S8 Table DMRs between severe SS cases and mild SS cases. Statistically significant DMRs (*fwerArea* ≤ 0.05) and their list of CpG sites. Here “region” refers to the location of the DMR relative to its closest gene, “value” is the average linear regression coefficients across CpG sites of a DMR, “area” is the absolute value of sum of linear regression coefficients for CpG sites of a DMR, “fwerArea” is the proportion of bootstraps with at least one candidate DMR area greater than observed DMR area, and “p.valueArea” is proportion of bootstraps with maximum bump area exceeding the observed area. For CpG site “island location”, “N_Shore” = north shore, “S_Shore” = south shore, “N_Shelf” = north shelf, and “S_Shelf” = south shelf, and “OpenSea” = open sea.
(XLSX)

S1 File (DOCX)
