# Supplementary figures and images for: Identification of Sjögren’s syndrome patient subgroups by clustering of labial salivary gland DNA methylation profiles
Source: PLoS One. 2023 Mar 2;18(3):e0281891. doi: 10.1371/journal.pone.0281891 (PMC9980741; doi:10.1371/journal.pone.0281891)

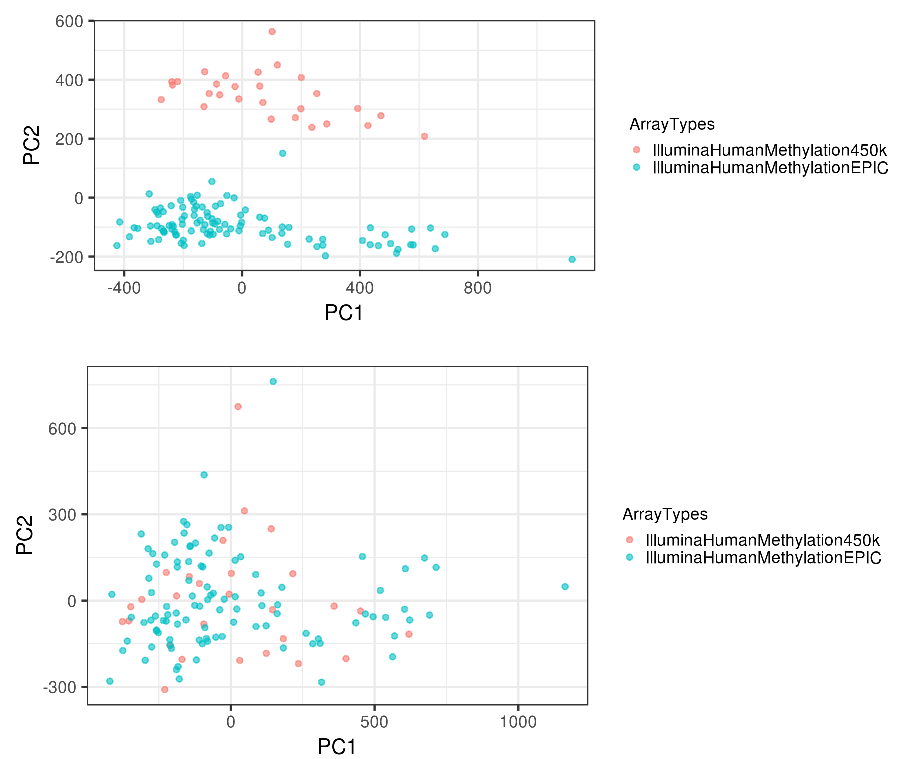

Supplement: S1 Fig — The DNA methylation array (i.e. 450K or EPIC) is indicated by color. PC2 captures variation in DNA methylation explained by array type. (A) Before batch-correction. (B) After batch-correction. (TIF) [file pone.0281891.s001.tif]

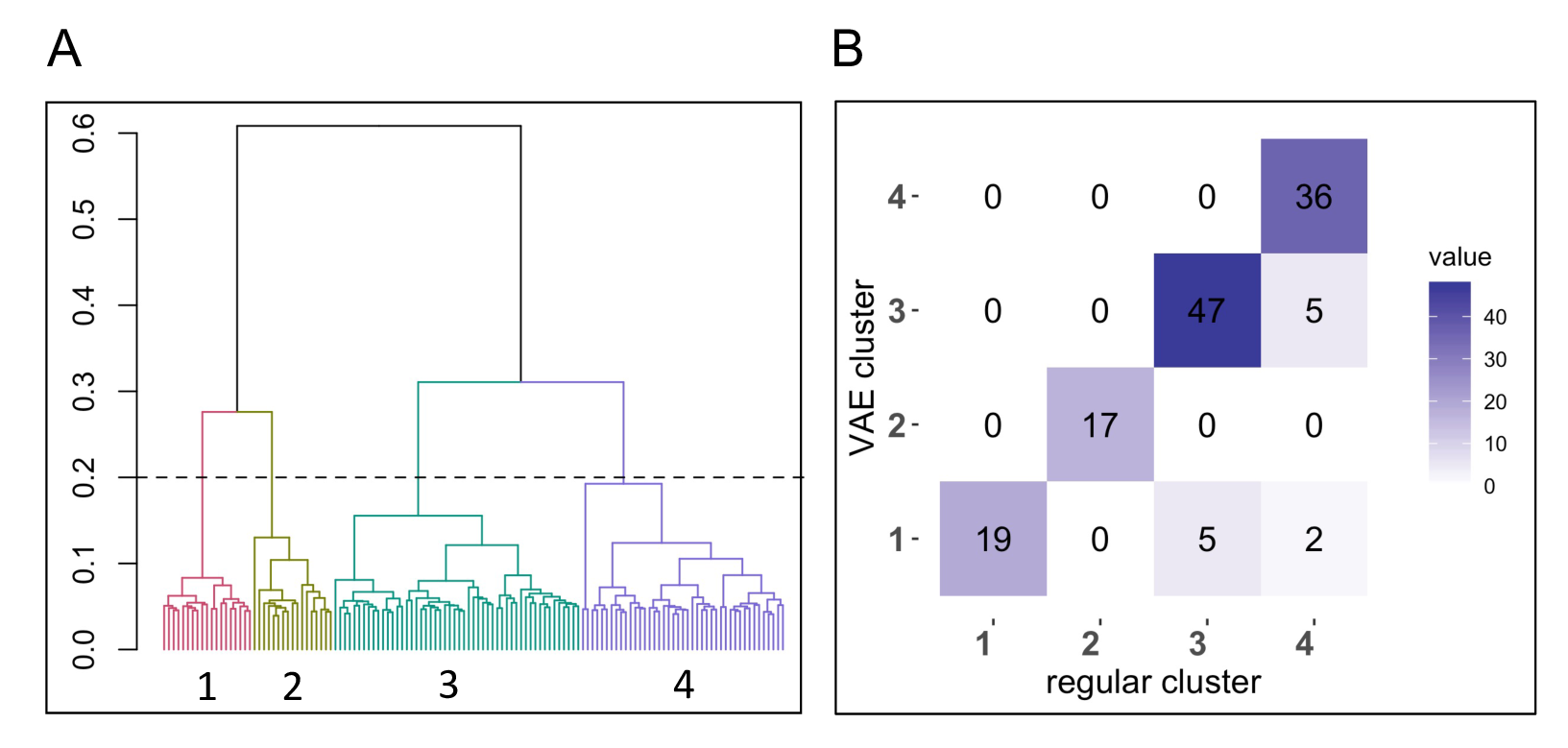

Supplement: S2 Fig — (A) Dendrogram of baseline hierarchical clustering of DNA methylation profiles (see Materials and methods). (B) Confusion matrix showing agreement of clustering results between the baseline approach and VAE-based approach (Fig 1). (TIFF) [file pone.0281891.s002.tiff]

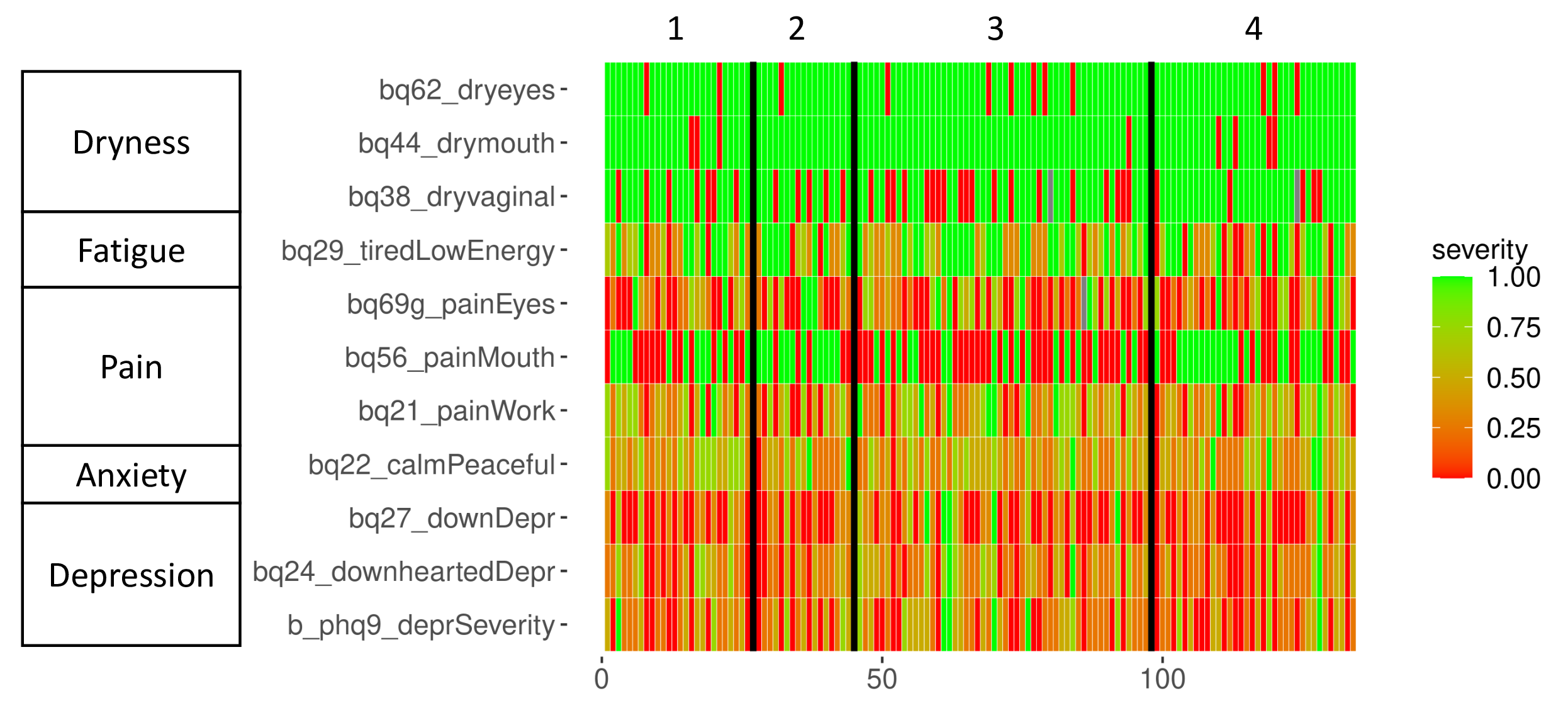

Supplement: S3 Fig — All phenotypes are either ordinal or binary, and normalized between 0 and 1, with larger values indicative of greater severity. Clinical phenotypes are grouped by general categories of dryness, fatigue, pain, anxiety, and depression. Each column represents a patient and all 131 subjects are grouped by patient clusters. Gray indicates missingness. See S4 Table for clinical phenotype key. (TIFF) [file pone.0281891.s003.tiff]

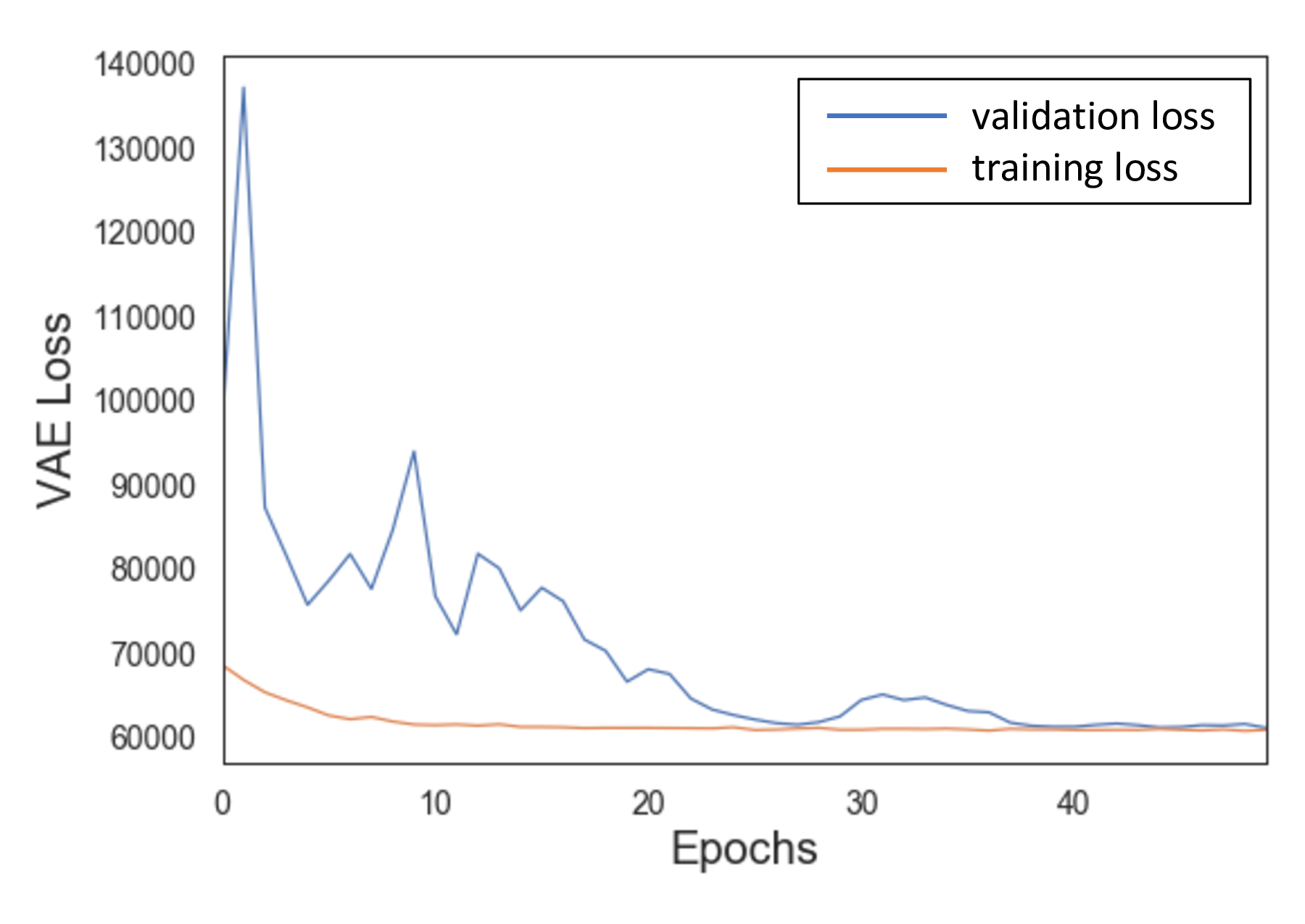

Supplement: S5 Fig — (TIFF) [file pone.0281891.s005.tiff]
