# Supplementary material for: Identification of Sjögren’s syndrome patient subgroups by clustering of labial salivary gland DNA methylation profiles
Source: PLoS One. 2023 Mar 2;18(3):e0281891. doi: 10.1371/journal.pone.0281891 (PMC9980741; doi:10.1371/journal.pone.0281891)
Supplement: S1 File — (DOCX) [file pone.0281891.s014.docx]

**Supplementary Text**

**Supplementary Materials and Methods**

**Variational autoencoder summary**. We provide a brief review of VAE, and refer to Kingma and Welling for complete details[1]. Given an input dataset $\boldsymbol{X}=\{x_{i}{\}}_{i=1}^{n}$ where $x_{i}\in\mathbb{R}^{p}$ (in our case $p$ is the number of CpG sites), the VAE learns a distribution of latent variables $z\in\mathbb{R}^{m}$ where $m<p$. Let $q_{\phi}(z|x)$ denote the latent variable distribution specified by an encoder with parameters $\phi$, and let $p_{\theta}(x|z)$ denote the output distribution from a decoder with parameters $\theta$. Then, the VAE method maximizes a lower bound of the log likelihood known as the evidence lower bound (ELBO)

|  | $\log p_{\theta}\left( x \right)\geq\mathbb{E}_{q_{\phi}(z\vert x)}[\log p_{\theta}(x\vert z)]-D_{KL}(q_{\phi}\left( z \vert x \right)\parallel p(z)),$ | (1) |
| --- | --- | --- |

where $D_{KL}(q_{\phi}\left( z | x \right)\parallel p\left( z \right))$ is the Kullback-Leibler (KL) divergence between distributions $q_{\phi}\left( z | x \right)$ and $p\left( z \right)$. The distribution $p\left( z \right)$is chosen to be the standard multivariate normal distribution $\mathcal{N}(0, I)$. The ELBO terms have straightforward interpretations—maximizing the first term minimizes the reconstruction loss and minimizing the KL divergence constrains the latent variable distribution to be close to $p\left( z \right)$. Thus, $q_{\phi}\left( z | x \right)$ is chosen to belong to the multivariate normal distribution family with diagonal covariance, and the encoder estimates the mean and variance terms that specify the distribution. Reconstruction is learned by minimizing average binary cross entropy between $p_{\theta}(x|z)$ and input β-values, where $p_{\theta}(x|z)$ is chosen to be sigmoid activation for each CpG site.

The choice of $p\left( z \right)$ as a standard multivariate normal allows the VAE to learn latent variables with desirable properties. These properties are (1) statistical independence of latent variables and, depending on the decoder, (2) smoothness of the latent space[2]. In other words, for (2), interpolation in the latent space corresponds to interpolation in the feature space of the data. For our application of clustering in this latent space, the smoothness property is important because distances in the latent space better reflects differences in DNA methylation profiles.

We used the VAE implementation *Tybalt* and its hyperparameters[3], with a few exceptions. In particular, we trained with a batch size of 16 and a maximum of 50 epochs. We used the means outputted by the encoder as latent features of methylation data. Following guidelines from Way and Greene[3], the entire dataset was split into a 9:1 train:validation ratio and the VAE was applied to the top 100,00 most variable CpG sites by median absolute deviation. Supplementary Figure 5 shows that both training and validation VAE loss converged after approximately 40 epochs, or iterations over the training dataset. Refer to Way and Greene[3] for additional implementation details.

**Gene set enrichment analysis.** The GO gene set totals 5,917, with 4,436 derived from biological process ontology, 580 from cellular component ontology, and 901 from molecular function ontology. Additionally, we included two gene sets consisting of genes shown to be differentially methylated or differentially expressed respectively, between SS cases and controls in LSG[4,5]. We eliminated large gene sets numbering more than 100 genes for improved specificity GSEA results, retaining approximately 76% of gene sets. Since genes in the same pathway tend to be up or down-regulated together[6], We report the top 10 enrichment results by statistical significance as sufficient to provide an overall biological picture, and avoid interpreting the rest of the results, since GSEA with the hypergeometric test makes unrealistic independence assumptions between genes[7].

**Supplementary References**

1. Kingma DP, Welling M. Auto-encoding variational bayes. In: 2nd International Conference on Learning Representations, ICLR 2014 - Conference Track Proceedings. International Conference on Learning Representations, ICLR; 2014.

2. Higgins I, Matthey L, Glorot X, Pal A, Uria B, Blundell C, et al. Early Visual Concept Learning with Unsupervised Deep Learning. 2016 Jun 17 [cited 2020 Jun 9]; Available from: http://arxiv.org/abs/1606.05579

3. Way GP, Greene CS. Extracting a biologically relevant latent space from cancer transcriptomes with variational autoencoders. In: Pacific Symposium on Biocomputing. World Scientific Publishing Co. Pte Ltd; 2018. p. 80–95.

4. Cole MB, Quach H, Quach D, Baker A, Taylor KE, Barcellos LF, et al. Epigenetic Signatures of Salivary Gland Inflammation in Sjögren’s Syndrome. Arthritis Rheumatol (Hoboken, NJ). 2016;68(12):2936–44.

5. Hjelmervik TOR, Petersen K, Jonassen I, Jonsson R, Bolstad AI. Gene expression profiling of minor salivary glands clearly distinguishes primary Sjögren’s syndrome patients from healthy control subjects. Arthritis Rheum. 2005 May;52(5):1534–44.

6. Hong G, Zhang W, Li H, Shen X, Guo Z. Separate enrichment analysis of pathways for up- and downregulated genes. J R Soc Interface. 2014 Mar 6;11(92):20130950.

7. Tamayo P, Steinhardt G, Liberzon A, Mesirov JP. The limitations of simple gene set enrichment analysis assuming gene independence. Stat Methods Med Res. 2016 Feb 14;25(1):472–87.

8. Li H, Reksten TR, Ice JA, Kelly JA, Adrianto I, Rasmussen A, et al. Identification of a Sjögren’s syndrome susceptibility locus at OAS1 that influences isoform switching, protein expression, and responsiveness to type I interferons. PLoS Genet [Internet]. 2017 [cited 2020 Jun 26];13(6). Available from: https://pubmed.ncbi.nlm.nih.gov/28640813/

9. Taylor KE, Wong Q, Levine DM, McHugh C, Laurie C, Doheny K, et al. Genome-Wide Association Analysis Reveals Genetic Heterogeneity of Sjögren’s Syndrome According to Ancestry. Arthritis Rheumatol. 2017 Jun 1;69(6):1294–305.

10. Lessard CJ, Li H, Adrianto I, Ice JA, Rasmussen A, Grundahl KM, et al. Variants at multiple loci implicated in both innate and adaptive immune responses are associated with Sjögren’s syndrome. Nat Genet. 2013 Nov;45(11):1284–94.

11. Liberzon A, Birger C, Thorvaldsdóttir H, Ghandi M, Mesirov JP, Tamayo P. The Molecular Signatures Database Hallmark Gene Set Collection. Cell Syst. 2015 Dec 23;1(6):417–25.
